# Editorial: Intestinal epithelial barrier disruption by enteric pathogens

**DOI:** 10.3389/fcimb.2023.1134753

**Published:** 2023-01-13

**Authors:** Arun K. Bhunia, Rana Al-Sadi

**Affiliations:** ^1^ Molecular Food Microbiology Laboratory, Department of Food Science, Purdue University, West Lafayette, IN, United States; ^2^ Purdue University Interdisciplinary Life Science Program (PULSe), West Lafayette, IN, United States; ^3^ Purdue Institute of Inflammation, Immunology and Infectious Disease, Purdue University, West Lafayette, IN, United States; ^4^ Department of Comparative Pathobiology, Purdue University, West Lafayette, IN, United States; ^5^ Department of Medicine, Division of Gastroenterology and Hepatology, Penn State College of Medicine, Hershey, PA, United States

**Keywords:** intestinal epithelial barrier, tight junction, enteric pathogen, gut microbiota, dysbiosis, inflammation

The intestinal epithelium provides a physical barrier that separates the many trillions of intestinal microbiota in the intestinal lumen from the underlying lamina propria and the deeper intestinal layers. A defective intestinal epithelial tight junction (TJ) barrier, characterized by an increase in intestinal permeability, is an important factor contributing to the pathophysiology of some enteric bacteria and other physiological assaults. It has been demonstrated that some enteric pathogens can induce an increase in intestinal epithelial TJ permeability, mediated by their virulence proteins or toxins, by allowing paracellular translocation of pathogens or permeation of luminal antigens that elicit and promote an inflammatory response. Understanding the complex interaction between intestinal microbiota (commensal and pathogenic) and the host intestinal epithelial TJ barrier may provide crucial insights into the pathogenesis of gut inflammation as well as therapeutic interventions to prevent intestinal epithelial barrier dysfunction and subsequently reduce the development of intestinal inflammation.

This Research Topic, “*Intestinal epithelial barrier disruption by enteric pathogens*”, invited articles that report the molecular basis for enteric pathogens, modulation of intestinal barrier function, regulation of host physiology and cellular function; *in vitro* and *in vivo* model systems for enteric pathogenesis, signaling pathways induced by innate immune receptors demonstrating the active role of intestinal epithelial TJ barrier in the host-microbial interplay; clinical research on the mucosal interaction between host and microbes contributing to development in intestinal and systemic inflammation; and studies investigating interventions or solutions to eliminate enteric pathogens, strengthen epithelial barrier function, or block enteric pathogens-induced intestinal inflammation. There were 5 published articles contributed by 31 authors addressing insights into complex microbial interaction with the host intestinal environment leading to barrier dysfunction, diarrhea, acute pancreatitis, necrotizing enterocolitis, and colorectal anastomosis leak.

Su, et al. reviewed postweaning diarrhea in piglets and a probiotics-mediated approach to controlling such episodes. Post-weaning diarrhea not only increases mortality but also affects piglet health resulting in huge economic losses to the farmers. Besides physiological, environmental, and social changes during weaning, microbial agents also contribute to piglet diarrhea. The primary pathogens of concern are rotavirus, enterotoxigenic *E. coli*, and *Salmonella enterica*. Nutritional imbalance, stress, and anxiety due to separation from the mother during weaning disturb the gut microbiota homeostasis, and immunological imbalance leads to gut barrier dysfunction. Crucially, microbial infection exacerbates the barrier dysfunction thus antibiotics are routinely used as a remedy. In the current landscape, antibiotics use is not a sustainable solution therefore alternative approaches including probiotics are used not only to disrupt colonization or inactivate pathogens but also to improve immunomodulatory response by recruiting regulatory T cells but also inducing the secretion of anti-inflammatory cytokines, such as IFN-γ, IL-4, and IL-10.

Microbial metabolic byproducts, especially the short-chain fatty acids exert positive beneficial effects on intestinal epithelial cells leading to immune homeostasis, gut health, and well beings. In contrast, some intermediate metabolic by-products may adversely affect intestinal barrier function and negatively impact health leading to colitis and other inflammatory diseases. Yan et al. examined the production of succinate, a metabolic signaling molecule, and its contribution to necrotizing enterocolitis (NEC) in preterm infants in their original research report. They observed elevated levels of succinate in feces from NEC patients than those of non-NEC patients thus they were motivated to investigate the role of microbiota in succinate production and the onset of NEC. Increased succinate production in NEC neonates is attributed to a higher abundance of *Enterobacteriaceae* and a lower abundance of *Lactobacillaceae* and *Lactobacillus*. Likewise, in murine pups, the increased abundance of *Clostridiaceae*, *Enterococcaceae*, *Clostridium*_sensu_stricto_1, and *Enterococcus*, while the decreased abundance of *Lactobacillaceae* and *Lactobacillus* are responsible for increased levels of succinate. Elevated succinate levels were also linked to reduction of body weight gain, dysfunction of intestinal TJ barrier, upregulation of inflammatory cytokines production (TNFα, IL-1β, IL-6, and IL-18) and downregulation of anti-inflammatory cytokines, IL-10 and TGF-β. Exogenous succinate also increased expression of succinate receptor 1 (SUCNR1) and hypoxia-inducible factor 1a (HIF-1a), indicating that the activation of the HIF-1a signaling pathway might be required for NEC disease progression and disease severity.

Gut microbiota dysbiosis and impaired intestinal TJ barrier function are thought to contribute to acute pancreatitis. Bacterial cell wall peptidoglycan components such as diaminopimelic acid (DAP) are hypothesized to contribute to acute pancreatitis; however, the mechanism is poorly understood. DAP is a specific ligand for the cytoplasmic toll-like receptor, NOD1 (nuclear oligomerizing domain 1), and regulates NOD1/RIP2/NF-κB signaling pathway. Here, Jiao et al investigated the role of DAP in the crosstalk between the gut microbiota and pancreas during the onset of pancreatitis in an *in-vivo* rat model. Microbial community-derived DAP upregulated NOD1/RIP2/NF-kB and elevated serum DAP. Treatment of rats with neomycin or Chinese medicine, Qingyi Keli reduced DAP-induced inflammation and tissue damage by selectively inhibiting enteric bacteria, such as *Helicobacter* and *Lactobacillus*, without inhibiting the desirable bacteria including *Romboutsia* and *Allobaculum*. This study suggests that the gut microbiota-DAP-NOD1/RIP2 signaling pathway possibly plays an important role in the progression of acute pancreatitis, which can be prevented by early intervention by manipulating gut microbiota.

Colorectal anastomosis leak is potentially fatal complications following colorectal surgery, for which gut microbiota dysbiosis has been attributed to be one of the risk factors. However, the exact mechanism of dysbiosis is poorly understood. In the review article, Liu et al. thoroughly examined how microbiota play a role in the onset of anastomotic leak. They revealed that anastomotic leak-specific pathogens are enriched on the anastomosis site resulting in tissue breakdown and intestinal barrier damage. Based on their in-depth literature analysis, they proposed three-tiered events that lead to anastomosis leak in patients undergoing colorectal surgery; (i) shaping the gut microbiota by preoperative intervention, (ii) enrichment of anastomotic leak-specific pathogens with strong collagenase activity, biofilm formation and adhesive properties, and (iii) poor anastomotic healing. Shifting microbial community to a more desirable population during preoperative preparation may reduce dysbiosis and promote healing of colorectal anastomosis leak.

*Vibrio cholerae* is a prototype enteric pathogen that can effectively overcome host innate defense, colonize the intestine, promote intestinal TJ barrier dysfunction, and induce profuse watery diarrhea. A study by Cho et al investigated the role of gut microbiota in susceptibility to *V. cholerae* infection in a suckling mice model that was implanted with microbiota from humans susceptible to recurrent diarrhea and malnutrition in cholera endemic areas, or cholera-resistant healthy individual. *Vibrio*-infected animals receiving microbiota from susceptible hosts exhibited downregulation of genes responsible for reactive oxygen/nitrogen stress. Interestingly, biofilm-associated genes (*vpsL*) were upregulated in *V. cholerae* and the bacterium is more resistant to oxidative stress, and bile metabolites generated by the increased activity of bile salt hydrolase produced by the commensal microbes. This study unraveled a unique contribution of commensal microbes in enteric pathogen colonization and resistance to host factors thereby exacerbating disease severity and infection in a susceptible host.

The articles published in this Research Topic enhanced our understanding of enteric pathogen-induced gut barrier dysfunction, which is often exacerbated by microbiota-induced dysbiosis. The authors are sincerely acknowledged for their outstanding contributions to making this collection a great success.

## Author contributions

AB prepared the draft. RA-S contributed to editing. Both AB and RA-S approve submission.

